# Early puberty in 11-year-old girls: Millennium Cohort Study findings

**DOI:** 10.1136/archdischild-2016-310475

**Published:** 2016-09-26

**Authors:** Yvonne Kelly, Afshin Zilanawala, Amanda Sacker, Robert Hiatt, Russell Viner

**Affiliations:** 1 Department of Epidemiology and Public Health, University College London, London, UK; 2 Department of Epidemiology and Biostatistics, University of California San Francisco, San Francisco, California, USA; 3 Institute of Child Health, University College London, London, UK

**Keywords:** inequalities, ethnicity, socioeconomic, menarche

## Abstract

**Objective:**

Early puberty in girls is linked to some adverse outcomes in adolescence and mid-life. We address two research questions: (1) Are socioeconomic circumstances and ethnicity associated with early onset puberty? (2) Are adiposity and/or psychosocial stress associated with observed associations?

**Design:**

Longitudinal data on 5839 girls from the UK Millennium Cohort Study were used to estimate associations between ethnicity, family income, adiposity and psychosocial stress with a marker of puberty.

**Main outcome measure:**

Reported menstruation at age 11 years.

**Results:**

All quoted ORs are statistically significant. Girls in the poorest income quintile were twice as likely (OR=2.1), and the second poorest quintile nearly twice as likely (OR=1.9) to have begun menstruation compared with girls in the richest income quintile. Estimates were roughly halved on adjustment for Body Mass Index and markers of psychosocial stress (poorest, OR=1.5; second poorest, OR=1.5). Indian girls were over 3 times as likely compared with whites to have started menstruation (OR=3.5) and statistical adjustments did not attenuate estimates. The raised odds of menstruation for Pakistani (OR=1.9), Bangladeshi (OR=3.3) and black African (OR=3.0) girls were attenuated to varying extents, from about a third to a half, on adjustment for income and adiposity.

**Conclusions:**

In contemporary UK, excess adiposity and psychosocial stress were associated with social inequalities in early puberty, while material disadvantage and adiposity were linked to ethnic inequalities in early puberty among girls.

**What is already known on this topic?**

- Early onset puberty is linked to some aspects of poor well-being and health throughout the life course.
- Prior studies, mostly from the USA, have examined potential underlying causes of early puberty but these are not well understood.

**What this study adds?**

- In contemporary UK, girls from socioeconomically disadvantaged groups and some ethnic minority groups are most likely to have early onset puberty.
- Excess adiposity and psychosocial stress among girls from low income backgrounds was associated with early onset puberty.
- Material disadvantage and adiposity were associated, to varying degrees, with early puberty in girls from Pakistani, Bangladeshi and black African backgrounds.

## Introduction

Early onset puberty in girls is linked to certain adverse outcomes during different stages of the life course, as it is associated with the increased risk of early sexual activity and teenage pregnancy,^1^ poor mental health in adolescence^2^ ^3^ and mid-life cardiovascular disease,^4^ and breast cancer,^5^ but is associated with better bone health in early old age.^6^ The age of puberty onset decreased dramatically over the 20th century in high income countries with similar secular trends still evident in low and middle income countries.^7^ ^8^ It remains controversial whether there are ongoing secular trends in the timing of puberty in high income country settings.^8^ Prior studies, mostly from the USA, have described socioeconomic and ethnic/racial patterning of the onset of puberty.^9–14^ Given the different historical and geographical patterns of migration to the UK and USA we might not necessarily expect to see ethnic differences in early puberty in the UK. However, until now, work from the UK has not been able to examine ethnic differences across a representative range of ethnic minority groups, either because study samples lack diversity^15^ ^16^ or they focus on a single minority group.^16^ ^17^

Reported influences on the timing of puberty in girls include prenatal and postnatal growth and adiposity,^11^ ^16^ ^19–22^ psychosocial stress,^23^ ^24^ environmental pollutants,^25^ migration^26^ and genetic factors.^27^ We know that socioeconomic circumstances and ethnicity are linked to increased adiposity^28^ ^29^ and psychosocial stress.^30–32^ However, evidence from the small number of studies in different country settings on whether excess adiposity and psychosocial stress among socioeconomically disadvantaged and ethnic minority groups explains observed patterns of puberty onset is mixed.^10^ ^11^ ^17^

Comparison of study findings is sometimes hindered as studies use different markers of puberty. The accurate estimation of different stages of puberty, particularly in population surveys, is challenging. The most widely used indicators in girls are breast development and menstruation; the former difficult to distinguish unless by physical examination; the latter a unique event, regarded internationally, as the gold standard in clinical and population study settings.^7^ ^8^ In the UK, according to the most recent reference data, the median age for menstruation is 12.9 years.^33^

To our knowledge, this paper is the first to look longitudinally at two potential mechanisms linking socioeconomic position and ethnicity to early onset puberty in a contemporary UK setting. In a large-scale population based study of 11-year-old girls we investigate two research questions: (1) Are socioeconomic circumstances and ethnicity associated with early onset puberty? (2) Are excess adiposity and/or psychosocial stress associated with observed associations?

## Methods

The Millennium Cohort Study (MCS) is a UK nationally representative prospective cohort study of children born into 19 244 families between September 2000 and January 2002.^34^ Participating families were selected from a random sample of electoral wards with a stratified sampling design to ensure adequate representation of all four UK countries, disadvantaged areas and ethnically diverse areas. The first sweep of data was collected when cohort members were around 9 months and the subsequent four sweeps of data were collected at ages 3 years, 5 years, 7 years and 11 years. At all data collection sweeps interviews were conducted and anthropometric measurements were taken during home visits. During interviews cohort members' carers were asked about socioeconomic circumstances and the family psychosocial milieu; and at the age 11 years sweep mothers were asked questions about the onset of puberty. Interview data were available for 69% of families when cohort members were aged 11 years. Permissions were granted for MCS by relevant ethical committees at each sweep of data collection.

### Puberty

Data on puberty were mother reported using an adapted question from the Petersen Pubertal Development Scale: ‘Has she begun to menstruate (we mean started to have her period)?’, responses were: no (n=5247), yes (n=592), don't know/don't wish to answer (n=89), with participants in the latter category omitted from analysis. There was some variation in the proportion of participants with ‘don't know/don't wish to answer’ responses, ranging from richest (0.7%) to poorest income quintile (2.7%) and was lowest in the white (0.8%) and highest in the Bangladeshi origin group (8.4%).

### Socioeconomic background

Quintiles of equivalised family income at age 5 years were used as a marker of early life socioeconomic circumstances. This measure takes into account the size and composition of the household.^34^ ^35^ The richest quintile was used as the reference category in analysis.

### Ethnicity

All children were born in the UK and we used mothers’ reports of her child's particular ethnic origin (collected at age 9 months or 3 years, using UK census categories). For brevity ethnic origin categories are hitherto referred to as follows: white, Indian, Pakistani, Bangladeshi, black Caribbean (including mixed white and black Caribbean), black African (including mixed white and black African) and other (includes mixed ethnic groups and ethnic minority groups that could not be categorised into any of the otherwise defined groups). As the largest ethnic group, white was used as the reference category.

### Explanatory factors

At age 7 years children were weighed without shoes or outdoor clothing using Tanita BF-522W scales (Tanita UK Middlesex, UK). Weight in kilograms to one decimal place and per cent body fat were recorded. Heights were obtained using the Leicester Height Measure Stadiometer (Seca, Birmingham, UK) and recorded to the nearest millimetre. These measures were used to calculate Body Mass Index (BMI, (lean mass+fat mass)/height^2^) and Fat Mass Index (FMI, fat mass/height^2^). BMI and FMI were used in statistical models as markers of adiposity. Birth weight (kg), reported by mothers when cohort members were 9 months of age, was included as a marker of fetal growth in models.

Markers of psychosocial stress, collected at age 7 years, were: mother's psychological distress (Kessler-6 Score),^36^ lone parent family (yes/no), child's socioemotional difficulties (total score using mother reported Strengths and Difficulties Questionnaire)^37^ and frequency of racist attacks/insults in residential area (not common vs fairly or very common). Due to data availability this latter marker was from the age 5 years data collection.

### Study sample

We analysed data on singleton-born cohort members for whom data on menstruation were available. The analytical sample was 5839 after multiply imputing missing values on explanatory factors. The amount of missing data ranged from 0% to 18%. We used multiple imputation techniques, which account for uncertainty about missing values by imputing several values for each missing data point.^38^ We imputed 25 data sets, consolidated results from all imputations using Rubin's combination rules^39^ and excluded cases with imputed values on menstruation from the analytical sample to improve the efficiency of estimates.^40^ Results from the imputed analyses did not vary substantively from the analyses using listwise deletion (analysis not shown).

### Analytical approach

We hypothesised that any income or ethnic patterning of puberty by age 11 years would be explained in part by adiposity and in part by psychosocial stress. In keeping with the hypothesised temporal sequence, we used family income at age 5 years to reflect early life socioeconomic circumstances, ethnicity collected from infancy, and to test potential mechanisms, markers of adiposity and psychosocial stress (where possible) were measured at age 7 years. Birth weight as a marker of prenatal growth which has been linked to early puberty onset^19^ was used as a control variable. Logistic regression modelling was used to estimate associations.

- Model 0 is the baseline model and shows age (centred) adjusted estimates for income and ethnicity.
- Model 1 simultaneously adjusts for age, income and ethnicity.
- Model 2 is model 1 plus birth weight.
- Model 3 is model 1 plus a marker of adiposity (BMI or FMI).
- Model 4 is model 1 plus markers of psychosocial stress.
- Model 5 is a combination of models 2–4, simultaneously adjusting for all variables.

We tested for effect modification by running models containing interaction terms for ethnicity by income but none of these interactions were statistically significant. All analysis was carried out using Stata V.13.1 (Stata). Analyses used sample weights to adjust for the unequal probability of being sampled and the stratified and clustered sample design.

## Results

The average age of girls was 11.2 years (SD=0.33) and 9.5% had begun menstruation. Menstruation was socially graded (14.1% of poorest quintile and 6.8% in richest, p<0.001). Indian, Bangladeshi and black African girls were most likely (24.2%, 21.6% and 20.1%, respectively) to have begun menstruation (table 1).

**Table 1:** Proportions of girls menstruating (weighted %), by income and ethnicity

	Per cent	Sample size
Overall	9.5	5839
Income quintile		
Richest	6.8	1115
Fourth	7.2	1163
Third	8.8	1156
Second	12.8***	1200
Poorest	14.1***	1205
Ethnic group		
White	8.6	4989
Indian	24.2***	129
Pakistani	14.8**	239
Bangladeshi	21.6***	109
Black Caribbean	13.0	126
Black African	20.1**	117
Other	15.3*	130

Significant differences are in reference to richest quintile for income and to white for ethnicity. Unweighted sample sizes are conditional on observed ethnicity and mother reported menstruation.

***p<0.001, **p<0.01, *p<0.05.

On average, girls who had begun menstruation were more adipose at age 7 years and were more likely to have been exposed to psychosocial stress earlier in childhood (table 2). BMI and FMI were higher for girls who had begun menstruation (BMI 17.6 kg/m^2^ vs 16.5 kg/m^2^; FMI 5.1 kg/m^2^ vs 4.2 kg/m^2^) compared with those who had not. Girls who had begun menstruation had mothers who had had higher psychological distress scores (3.6 vs 3.0), were more likely to be from a lone parent family (23.1% vs 17.2%) and had themselves higher socioemotional difficulties scores earlier in childhood (7.5 vs 6.4).

**Table 2:** Adiposity and psychosocial stressors by menstruation (weighted means and %)

	Menstruation		
	No (n=5247)	Yes (n=592)	Overall (n=5839)
Birth weight (kg), mean	3.3***	3.2	3.3 (0.6)
BMI (kg/m^2^), mean	16.5***	17.6	16.6 (2.3)
FMI (kg/m^2^), mean	4.2***	5.1	4.3 (1.5)
Mother's psychological distress (0–24), mean	3.0*	3.6	3 (3.6)
Lone parent family, %	17.2*	23.1	17.8 (0.4)
Socioemotional difficulties (0–35), mean	6.4***	7.5	6.5 (4.9)
Racism in area is fairly/very common, %	5.6	7.9	5.8 (0.2)

SDs in parentheses.

***p<0.001, *p<0.05.

BMI, Body Mass Index; FMI, Fat Mass Index.

Table 3 shows regression estimates for the odds of menstruation across income and ethnic groups. Girls in the poorest income group were more than twice as likely (model 0: OR=2.14) and those in the second poorest group nearly twice as likely (model 0: OR=1.92) to have begun menstruation compared with girls in the richest income quintile. Estimates were attenuated on adjustment for BMI (model 3: poorest, OR=1.86; second poorest, OR=1.74), and on adjustment for markers of psychosocial stress (model 4: poorest, OR=1.60; second poorest, OR=1.64) but differences remained statistically significant in the fully adjusted model (model 5: poorest, OR=1.52; second poorest, OR=1.52).

**Table 3:** ORs (95% CI) for menstruation by income and ethnicity (n=5839)

	Model 0: age		Model 1: age, income, ethnicity	Model 2: Model 1+birth weight	Model 3: Model 1+BMI	Model 4: Model 1+psychosocial stressors	Model 5: fully adjusted
Income (ref.: richest quintile)							
Fourth	1.02 (0.7 to 1.5)		1.03 (0.7 to 1.5)	1.02 (0.7 to 1.5)	0.99 (0.7 to 1.5)	0.99 (0.7 to 1.5)	0.95 (0.6 to 1.4)
Third	1.27 (0.9 to 1.8)		1.29 (0.9 to 1.8)	1.28 (0.9 to 1.8)	1.26 (0.9 to 1.8)	1.21 (0.9 to 1.7)	1.18 (0.8 to 1.7)
Second	1.92*** (1.4 to 2.7)		1.86*** (1.3 to 2.6)	1.82*** (1.3 to 2.6)	1.74** (1.2 to 2.4)	1.64** (1.2 to 2.3)	1.52* (1.1 to 2.2)
Poorest	2.14*** (1.5 to 3.0)		1.95*** (1.4 to 2.8)	1.89*** (1.3 to 2.7)	1.86*** (1.3 to 2.7)	1.60* (1.1 to 2.4)	1.52* (1.0 to 2.3)
Ethnicity (ref.: white)							
Indian		3.53*** (2.2 to 5.8)	3.55*** (2.2 to 5.7)	3.26*** (2.0 to 5.2)	3.95*** (2.4 to 6.4)	3.69*** (2.3 to 6.0)	3.66*** (2.3 to 5.9)
Pakistani		1.87** (1.2 to 2.9)	1.43 (0.9 to 2.2)	1.35 (0.9 to 2.1)	1.62* (1.0 to 2.5)	1.39 (0.9 to 2.1)	1.45 (0.9 to 2.3)
Bangladeshi		3.27*** (2.2 to 5.0)	2.45*** (1.6 to 3.8)	2.24*** (1.4 to 3.5)	2.36*** (1.5 to 3.8)	2.54*** (1.6 to 4.0)	2.15** (1.3 to 3.5)
Black Caribbean		1.62 (0.9 to 3.1)	1.39 (0.7 to 2.6)	1.34 (0.7 to 2.5)	1.20 (0.6 to 2.4)	1.32 (0.7 to 2.5)	1.08 (0.5 to 2.2)
Black African		3.00*** (1.6 to 5.6)	2.53** (1.3 to 4.9)	2.50** (1.3 to 4.8)	1.88* (1.0 to 3.5)	2.58** (1.3 to 5.0)	1.87* (1.0 to 3.4)
Other		1.93* (1.0 to 3.7)	1.85 (1.0 to 3.5)	1.82 (0.9 to 3.5)	2.03* (1.0 to 4.0)	1.88 (1.0 to 3.6)	2.02 (1.0 to 4.1)
Age (in years and centred at mean)	4.73*** (3.5 to 6.4)	4.98*** (3.7 to 6.7)	4.87*** (3.6 to 6.6)	4.90*** (3.6 to 6.7)	4.81*** (3.5 to 6.5)	5.02*** (3.7 to 6.8)	4.97*** (3.6 to 6.8)
Birth weight (kg)				0.78** (0.6 to 0.9)			0.71*** (0.6 to 0.9)
BMI (kg/m^2^)					1.18*** (1.1 to 1.2)		1.19*** (1.1 to 1.2)
Mother's psychological distress						1.00 (1.0 to 1.0)	1.00 (1.0 to 1.0)
Racism in area is fairly/very common						1.16 (0.8 to 1.7)	1.03 (0.7 to 1.5)
Lone parent family						1.15 (0.9 to 1.5)	1.09 (0.8 to 1.5)
Total difficulties score						1.03** (1.0 to 1.1)	1.03* (1.0 to 1.1)

All estimates are weighted with overall survey weights.

***p<0.001, **p<0.01, *p<0.05.

BMI, Body Mass Index.

Adjusting for income (model 0 vs model 1) attenuated the raised odds of menstruation for Pakistani (1.97 vs 1.43), Bangladeshi (3.27 vs 2.45) and black African (3.00 vs 2.53) girls. Unlike the other ethnic minority groups adjustment for income did not reduce estimates for Indian girls due to their relative economic advantage (see online supplementary appendix 1). Adjustment for adiposity (model 3) further attenuated the estimated odds for Bangladeshi (2.36) and black African (1.88) girls, while adjusting for psychosocial factors (model 4) did little to change estimates. In fully adjusted models, differences remained statistically significant for Bangladeshi and black African girls (model 5: Bangladeshi, OR=2.15; black African, OR=1.87). Similar patterns of attenuation were observed when using FMI in models, but differences were no longer statistically different for the black African group (see online supplementary appendix table 2).

10.1136/archdischild-2016-310475.supp1 supplementary appendix table

## Discussion

In a large contemporary UK setting we found that 9.5% of girls aged 11 years had started menstruation as reported by their mothers. Girls from socioeconomically disadvantaged families and those from certain—Indian, Pakistani, Bangladeshi and black African—but not all ethnic minority groups were most likely to have begun menstruating. We found that for girls in the poorest two quintiles of the income distribution inequalities were roughly halved on adjustment for markers of adiposity and psychosocial factors but differences remained unexplained in final models. Because of the relatively advantaged position of families in the Indian group, our models did not attenuate the increased odds of early menstruation for these girls. In contrast, material disadvantage appeared to reduce the odds of early menstruation by about a half for Pakistani girls, and a combination of material disadvantage and higher adiposity among girls in the Bangladeshi and black African groups reduced the odds by about a third.

Our findings are comparable with data from the 1990 UK reference values which showed that 11.8% of girls had begun menstruation prior to leaving primary school,^32^ and this is consistent with the overall picture of no further declines in the timing of puberty in high income country settings.^7^ ^8^ There are important differences to note here though, as the Ten Towns study found no differences according to social position or ethnicity. However their marker of social position was crude and different from ours (manual vs non-manual occupational class), and they used aggregate ethnic minority groupings, ‘black’ and ‘South Asian’, which likely obscured important differences. Average age estimates for menstruation in low and middle income countries which may have some relevance for ethnic minority groups included in the current study are largely historical and limited in availability.^40^

Similar to our findings, socioeconomic gradients in puberty onset have been shown elsewhere,^12^ and two landmark studies suggested that disadvantaged early environments, characterised by some or all of poverty, family conflict, father absence and negative parenting were associated with early onset menstruation.^22^ ^23^ Other reports suggest that stressful circumstances just prior to puberty might have the opposite effect, delaying onset,^41^ ^42^ but findings from these reports are confounded by co-occurring nutritional stress which makes their interpretation difficult. Prior work has shown stark socioeconomic inequalities in psychosocial circumstances and risk of excess weight^27^ ^29^ lending support to their hypothesised role in early onset puberty among girls. However, our analyses suggest that markers of psychosocial stress such as having a mother with psychological distress, being in a one parent family and themselves having socioemotional difficulties at age 7 years were not independently predictive of early onset menstruation. In keeping with prior reports,^13–16^ ^18–21^ ^43^ we showed independent influences for markers of growth, with higher birth weight reducing the odds perhaps because low birthweight infants are more likely to exhibit catch up growth consistent with the increased risk of early puberty, and higher adiposity increasing the odds of early menstruation. Several studies report ethnic/racial differences in the occurrence of early onset puberty^9–13^ but proposed explanations vary according to minority group and by country setting. For example in the UK, in analyses of the Avon Longitudinal Study of Parents and Children non-white ethnicity is reported as a predictor of early onset puberty,^14^ while, Houghton *et al*^16^ have reported that the timing of menstruation for British-born Bangladeshi girls is comparable with that for whites. Studies that seek explanations for observed ethnic inequalities in health often find socioeconomic factors to be important,^28^ ^31^ ^44^ ^45^ but in relation to early onset puberty the picture is mixed. Similar to our observations for UK Pakistani, Bangladeshi and black African girls, Deardorff *et al*^10^ analysed US National Longitudinal Study of Youth data and found that the socioeconomic disadvantage of black and Hispanic girls compared with whites explained about half the disparity in early menarche. In contrast, Wu *et al*^46^ reported that socioeconomic disadvantage did not explain black-white differences in early onset menstruation, but that adjustment for BMI did reduce inequalities. Our findings highlight the different lived experiences of ethnic minority groups in the UK: for example, Indians are relatively advantaged whereas Pakistanis tend to be materially disadvantaged; Bangladeshis and black Africans are materially disadvantaged and tend to be more adipose compared with the majority ethnic group.

Our study has distinct strengths: we used data from a large-scale representative contemporary UK setting, and were able to examine associations across socioeconomic strata and the most common ethnic minority groups; we were able to investigate two hypothesised pathways—adiposity and psychosocial stress—which have been proposed to explain early onset puberty. On the other hand, our study has limitations. Girls’ menstruation was obtained from mother reports, this method of ascertainment has been reported to be reliable elsewhere,^13^ with no evidence of cultural or socioeconomic bias in the reporting of menarche. However, this cannot be entirely ruled out as even though the overall amount of missing data was small, there was some variation in the proportion of study participants in the ‘don't know/don't wish to answer’ category. As already noted we were able to investigate two hypothesised pathways and while our markers of adiposity were objectively measured, markers of the psychosocial environment were reported by the mother and could be prone to bias. There were a wider set of puberty markers available for use—breast development and pubic hair growth, but the reliability of these in population study settings is questionable and we chose not to use them. Due to lack of data we were not able to investigate the potential role of environmental pollutants. Nor were we able to examine the potential role of migration as all study participants were born in the UK; we did run analyses including mothers' migration status but this did not alter model estimates and had no independent relationship to menstruation in their daughters. Finally due to lack of data we were not able to take account of any heritable portion of early puberty. Taken together these limitations of data availability and imperfect measurement of environmental factors might help shed light on apparent economic and ethnic inequalities that remained after adiposity and psychosocial factors were taken account of.

Our findings and those of others’ highlight the complex and potentially opposing mechanisms at play for the onset of puberty.^7^ Future work using more complete prospective data on puberty from the same population sample will help to provide a more comprehensive picture of the timing of puberty in the UK. Data collections on girls with different migration histories and from a range of representative ethnic minority groups are needed to enhance understanding of the drivers of puberty in what are increasingly diverse populations.

## Conclusions

Socioeconomic and ethnic inequalities in early onset puberty are evident in the UK, and our findings suggest the unequal distribution of psychosocial stress and adiposity play a part for socioeconomic inequalities while material disadvantage and adiposity play a part in observed ethnic patterns. Socioeconomic and ethnic inequalities in later life health are evident, and early puberty could play a role in increasing chronic disease risk among women from disadvantaged groups. Given the short-term and long-term implications for early puberty on women's health and well-being, improving our understanding of underlying processes could help identify opportunities for interventions with benefits across the life course.
