# Supplementary material for: Early puberty in 11-year-old girls: Millennium Cohort Study findings
Source: Arch Dis Child. 2016 Sep 26;102(3):232–7. doi: 10.1136/archdischild-2016-310475 (PMC5339561; doi:10.1136/archdischild-2016-310475)
Supplement: supplementary appendix table [file archdischild-2016-310475supp_tables.pdf]

Appendix Table 1. Income distribution by ethnicity (%)

	White	Indian	Pakistani	Bangladeshi	Black Caribbean	Black African	Other
Equivalised household income							
Lowest quintile	13.4	16.7	47.8	55.5	37.0	40.1	21.1
Second quintile	17.1	18.6	32.5	24.9	20.2	21.2	19.1
Third quintile	21.5	13.0	8.9	12.8	11.5	16.2	13.7
Fourth quintile	22.7	24.8	2.8	0.5	17.7	9.9	18.7
Highest quintile	25.4	26.9	8.0	6.3	13.5	12.5	27.4

Notes. Sample size is 5,839. Percents are weighted by overall sample weights.

Appendix Table 2. Odds ratios (95% CI) for menstruation by income and ethnicity (n=5839)

	Model 0: age		Model 1: age, income, ethnicity	Model 2: Model 1 + Birthweight	Model 3: Model 1 + FMI	Model 4: Model 1 + psychosocial stressors	Model 5: Fully adjusted
Income (ref.: Richest quintile)							
Fourth	1.02		1.03	1.02	1.01	0.99	0.97
	(0.70 - 1.50)		(0.70 - 1.51)	(0.70 - 1.50)	(0.68 - 1.48)	(0.67 - 1.45)	(0.65 - 1.43)
Third	1.27		1.29	1.28	1.27	1.21	1.18
	(0.90 - 1.80)		(0.91 - 1.83)	(0.91 - 1.82)	(0.90 - 1.79)	(0.85 - 1.73)	(0.83 - 1.68)
Second	1.92***		1.86***	1.82***	1.77**	1.64**	1.55*
	(1.36 - 2.70)		(1.32 - 2.61)	(1.29 - 2.55)	(1.26 - 2.49)	(1.16 - 2.33)	(1.09 - 2.20)
Poorest	2.14***		1.95***	1.89***	1.91***	1.60*	1.56*
	(1.52 - 2.99)		(1.37 - 2.78)	(1.33 - 2.69)	(1.33 - 2.73)	(1.08 - 2.38)	(1.04 - 2.34)
Ethnicity (ref.: White)							
Indian		3.53***	3.55***	3.26***	3.92***	3.69***	3.58***
		(2.15 - 5.80)	(2.20 - 5.71)	(2.05 - 5.21)	(2.44 - 6.29)	(2.28 - 5.97)	(2.25 - 5.71)
Pakistani		1.87**	1.43	1.35	1.60*	1.39	1.42
		(1.23 - 2.85)	(0.94 - 2.18)	(0.88 - 2.07)	(1.03 - 2.49)	(0.91 - 2.15)	(0.90 - 2.25)
Bangladeshi		3.27***	2.45***	2.24***	2.31***	2.54***	2.07**
		(2.15 - 4.98)	(1.57 - 3.83)	(1.44 - 3.48)	(1.43 - 3.72)	(1.61 - 4.00)	(1.28 - 3.35)
Black Caribbean		1.62	1.39	1.34	1.12	1.32	0.99
		(0.85 - 3.06)	(0.74 - 2.62)	(0.71 - 2.54)	(0.55 - 2.26)	(0.69 - 2.49)	(0.47 - 2.08)
Black African		3.00***	2.53**	2.50**	1.65	2.58**	1.61
		(1.62 - 5.57)	(1.31 - 4.88)	(1.31 - 4.78)	(0.87 - 3.11)	(1.34 - 4.96)	(0.88 - 2.97)
Other		1.93*	1.85	1.82	2.00*	1.88	1.98
		(1.02 - 3.65)	(0.98 - 3.51)	(0.95 - 3.50)	(1.02 - 3.92)	(0.98 - 3.62)	(0.98 - 4.02)
Age (in years and centred at mean)	4.73***	4.98***	4.87***	4.90***	4.53***	5.02***	4.65***

	(3.52 - 6.36)	(3.69 - 6.73)	(3.61 - 6.57)	(3.62 - 6.65)	(3.31 - 6.20)	(3.72 - 6.76)	(3.38 - 6.39)
Birthweight (kg)				0.78**			0.69***
				(0.64 - 0.93)			(0.57 - 0.83)
FMI (kg/m^2)					1.31***		1.33***
					(1.24 - 1.39)		(1.26 - 1.42)
Mother's psychological distress						1.00	1.01
						(0.97 - 1.04)	(0.97 - 1.04)
Racism in area is fairly/very common						1.16	1.02
						(0.79 - 1.71)	(0.68 - 1.53)
Lone parent family						1.15	1.07
						(0.87 - 1.53)	(0.80 - 1.44)
Total difficulties score						1.03**	1.03*
						(1.01 - 1.05)	(1.00 - 1.05)

Notes: All estimates are weighted with overall survey weights.

*** p<0.001, ** p<0.01, * p<0.05
